# Impact of *KIT* exon 10 M541L allelic variant on the response to imatinib in aggressive fibromatosis: analysis of the desminib series by competitive allele specific Taqman PCR technology

**DOI:** 10.1186/1471-2407-14-632

**Published:** 2014-08-29

**Authors:** Armelle Dufresne, Laurent Alberti, Mehdi Brahmi, Sarah Kabani, Héloïse Philippon, David Pérol, Jean Yves Blay

**Affiliations:** Cancer Research Center of Lyon, INSERM UMR 1052, CNRS UMR 5286, Centre Leon Berard, 28 rue Laënnec, Lyon, France; Medical Oncology Department, Lyon, France; Biostatistics unit Anticancer Center Leon Berard, Lyon, France

**Keywords:** Aggressive fibromatosis, KIT exon 10 M541L allelic variant, Imatinib

## Abstract

**Background:**

Aggressive fibromatosis (AF) is a rare fibroblastic proliferative disease with a locally aggressive behavior and no distant metastasis, characterized by driver mutations in *CTNNB1* or the *APC* gene. When progressive and/or symptomatic AF is not amenable to local management, a variety of medical treatments may be efficient, including imatinib mesylate. The phase II “Desminib trial” included 40 patients with AF to evaluate the toxicity and efficacy of imatinib resulting in a 65% tumor control rate at 1 year. We investigated a potential predictive value of *KIT* exon 10 M541L variant (*KIT*^L541^) on this prospective series.

**Methods:**

DNA was extracted in sufficient quantity from 33 patients included in the Desminib trial. The detection of *KIT*^L541^ was performed by Competitive Allele-Specific Taqman® PCR technology. Chi-2 analyses were performed to search for a correlation between KIT status and tumor response. Progression free (PFS) and overall survival (OS) were compared by log-rank test after Kaplan-Meier analysis.

**Results:**

In 6 out of 33 cases (18%), the technique failed to determine the mutational status; 5 patients (19%) harboured *KIT*^L541^ and 22 patients (81%) were classified as *KIT* wild type. Compared with total cohort, *KIT*^L541^ frequency did not distinguish between different clinical characteristics. In the *KIT*^L541^ and the *KIT*^WT^ subgroups, the tumor control rate at 1 year was 100% and 68%, respectively (p = 0.316). The median PFS of patients harboring *KIT*^L541^ or not is 29.9 and 24.5 months, respectively (p = 0.616), and the median OS is not reached, in any of the groups.

**Conclusion:**

Our results do not support a predictive effect of *KIT*^L541^ on the efficacy of imatinib for patients with AF.

## Background

Aggressive fibromatosis (AF) is a rare fibroblastic proliferative disease characterized by driver mutations in *CTNNB1*, at specific sites of exon 3, or in the *APC* gene (in the context of Gardner syndrome). The management of AF has substantially evolved in the last 10 years [1]. AF are characterized by an aggressive local behavior, yet are unpredictable, with a risk of relapse after surgical excision but a lack of distant metastasis. These tumors are characterized by heterogeneity in their clinical presentation with an unpredictable clinical course. The classical strategy of aggressive front-line therapy with surgery and radiotherapy is now debated and a wait-and-see policy at initial presentation is often proposed (NCCN 2012 Guidelines) [2]. Systemic treatments such as non-steroid anti-inflammatory drugs (NSAIDs), hormonal treatment, cytotoxic chemotherapy, imatinib, or sorafenib are often used to control tumor growth and/or to relieve symptomatic AF, all with moderate and variable efficacy [3–8]. This observation raises the need to identify biomarkers, to effectively select patients who would benefit from a particular treatment.

In 2 prospective series of patients treated with imatinib, progression free survival (PFS) was 66% and 67% at 1 year [7, 8]. The phase II “Desminib trial” included 40 patients to evaluate the toxicity and efficacy of imatinib administered to patients with AF not amenable to radiotherapy or non-mutilating surgery. The results showed a disease control by imatinib in a large proportion of patients with 4 (10%) complete or partial confirmed responses and 28 (70%) with stable disease as best response, leading to a 1 year PFS of 67% [7].

*KIT* is one of the major targets of imatinib; mutations of *KIT* predict the efficacy of the drug in gastro intestinal stromal tumors (GIST) [9], but also in melanoma and thymic carcinoma [10, 11]. Several case reports have suggested a potential role of the *KIT* exon 10 M541L variant (*KIT*^L541^) in sensitivity of AF to imatinib [12, 13]. The present study was conducted on the Desminib series to search for a potential predictive value of *KIT*^L541^.

## Methods

### Patients

This study was performed as a retrospective translational research program on tumor samples of patients included in the Desminib trial [7]. Forty patients with progressive or recurrent AF that could not be treated with curative surgery or radiotherapy were included in the Desminib phase I/II trial to evaluate the efficacy and toxicity of imatinib. Patients with adequate end organ function were treated with 400 mg of imatinib daily, increasing to 800 mg in case of progressive disease. Best clinical response to imatinib was defined according to RECIST criteria. Evaluations were performed every 3 months. All evaluations of tumor responses to imatinib were reviewed by a radiological independent validation committee. Study investigations were carried out after approval by Lyon Ethics Committee (Comité Consultatif de Protection des Personnes se Prêtant à une Recherche Biomédicale, date of approval: 25 May 2004) and the French National Agency for Human Investigations (Agence Française de Sécurité Sanitaire des Produits de Santé, date of approval: 11 March 2004). Written informed consent was obtained from each patient to enroll them in the study and collect archival pathology specimens.

### Tissue samples

The analysis was performed on the initial tumors of patients, obtained by biopsy or surgical excision at the date of the diagnosis of the disease. Paraffin-embedded tissues samples of patients included in the study were obtained from pathology centers, all from tumors at initial diagnosis.

### DNA extraction

Total DNA was extracted from tumors using QIAamp DNA kit N° 56404 (Qiagen, France) according to the manufacturer’s instructions and quantified by spectrophotometry (NanoDrop ND-100 instrument, Thermo Fisher Scientific, Waltham, MA). Briefly, formalin-fixed paraffin-embedded (FFPE) tumors were lysed for 24 h in ATL buffer supplemented with proteinase K at 60°C in rotative agitation after washes with toluene and ethanol, in this order. Genomic DNA was isolated with a QIAamp MiniElute column.

### Competitive Allele-Specific Taqman® PCR (CAST-PCR)

**Figure 1:**
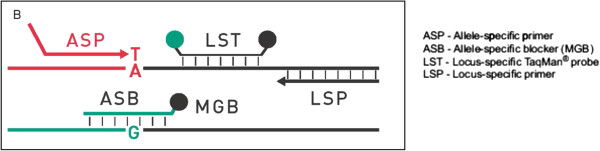
**In Competitive Allele-Specific Taqman® PCR technology, each mutant allele assay detects specific mutant alleles and a blocker suppresses the wild type allele.**

The detection of *KIT*^541^ status was performed by Competitive Allele-Specific Taqman® PCR technology provided by Applied Biosystems® (Figure 1). Each mutant allele assay detects specific mutant alleles. Each assay contains: an allele-specific primer that detects the mutant allele, an MGB oligonucleotide blocker that suppresses the wild type allele, a locus specific primer and a locus specific TaqMan® FAM™ dye-labeled MGB probe. Gene reference assays detect the genes that the target mutations reside in. They are designed to amplify a mutation-free and polymorphism-free region of the target gene. Each assay contains: a locus-specific pair of forward and reverse primers and a locus specific TaqMan® FAM™ dye-labeled MGB probe.

In a mutation detection experiment, a sample of unknown mutation status is run in individual real-time PCRs with one assay that targets mutant alleles within a gene and the corresponding gene reference assay. After amplification, the Ct (Cycle threshold) values of each mutant allele assay and the gene reference assay are determined by the Applied Biosystems® real-time PCR instrument software.

A mutation is detected in the DNA sample if Ct_mut_ < 38 AND Ct_rf_ < 35. If Ct_mut_ > 38 and/or Ct_rf_ > 35, the software classifies the gDNA sample as mutation not detected; the sample is either mutation negative, or below the limit of detection for the TaqMan® Mutation Detection Assays. Ct was also determined for exogenous IPC (Internal Passive Control) reagents added to each reaction to evaluate PCR failure or inhibition in a reaction.

### qPCR conditions

qPCR runs were performed in 96-well plates, in a final volume of 20 μL comprising 10 μL 2X Taqman Genotyping Mastermix (Applied Biosystems), 0.4 μL 500X Exogenous IPC template DNA, 2 μL 10X Exogenous IPC mix, 2 μL each primer (*KIT*^L541^ and Reference), 1.6 μL deionized water and 20 ng DNA (in 4 μL). Runs were performed on the ViiA™ 7 Real-Time PCR System using the following set of reaction conditions: 95°C ^10:00^ [92°C ^00:15^; 58°C ^01:00^] _5_ [92°C ^00:15^; 60°C ^01:00^] _40_.

### KIT^541^ validation

For 10 patients among the 33 patients tested by CAST-PCR, the determination of KIT exon 10 status was also determined by sequencing, using the method extensively described previously [14].

### Statistical analysis

Statistics were performed using R software. Chi-2 analyses were performed in order to study the distribution of known prognostic factors (age, tumor size and location) [15, 16] according to *KIT* status and in order to search for a correlation between *KIT* status and tumor response. PFS and OS of patients harboring or not *KIT*^L541^ variant were compared by log-rank test after Kaplan-Meier analysis.

## Results

DNA was obtained in sufficient quantity for 33 of the 40 patients included in the Desminib trial. Characteristics of these patients and their tumor samples are presented in Table 1. The clinical characteristics of patients are similar to those described in the literature, with a majority of female patients, a median age at diagnosis of 40, and patients presenting mainly large tumors. The FFPE blocks were taken between 7 to 15 years ago. Prognostic factors were well balanced between the 2 groups compared (patients with tumor harboring or not KIT^L541^) and therefore, could not influence the result.

**Table 1:** **Characteristics of the 33 patients and their FFPE samples analyzed (%)**

		Total	KIT ^L541^	KIT ^WT^
		n = 33	n = 5	n = 22
**Patients**				
Gender	Male	11 (33)		
	Female	22 (67)		
Median age at diagnosis				
[range], years		40 [20–72]	48 [39–57]	39 [20–72]
			Chi-2: p = 0,22	
Tumor location	Intra abdo	6 (18)	-	2 (9)
	Abdo wall	3 (9)	2 (40)	4 (18)
	Extra abdo	24 (73)	3 (60)	15 (68)
			Chi-2: p = 0,51	
Median tumor size [range], mm		100 [25–220]	70 [60–189]	92 [33–220]
			Chi-2: p = 0,44	
Familial Adenomatous Polyposis	Yes	5 (15)		
	No	28 (85)		
Performans status	0	22 (67)		
	1	8 (24)		
	2	1 (3)		
	Unknown	2 (6)		
Median TTP [range], months		24.6 [2.8-42.3]		
**FFPE samples**				
Blocks age	1997-1999	11 (33%)		
	2000-2005	22 (66%)		
Mean DNA quantity [range], ng/μl		782,14 [106,42-1748,86]		
Mean A260/280 ratio [range]		1,98 [1,76-2,05]		

**Figure 2:**
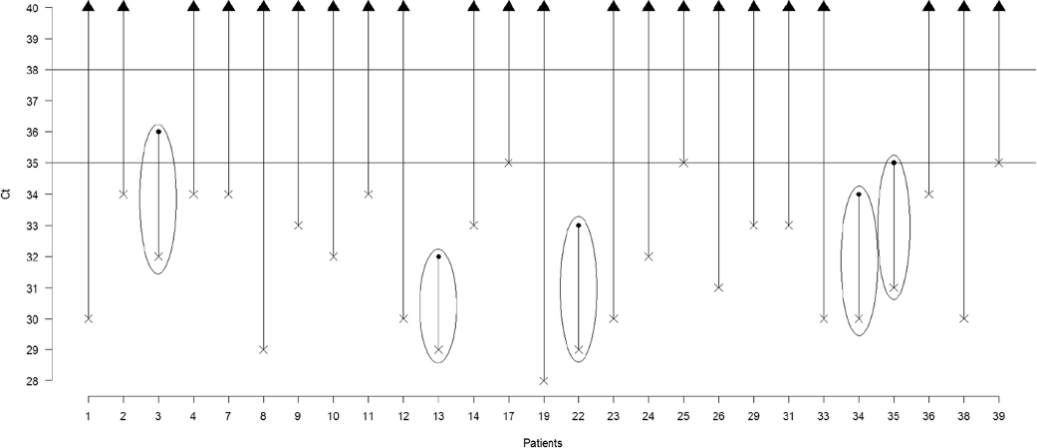
**For each evaluable patient, the cross represents Ct**_**rf**_ **and the point represents Ct**_**mut**_ **.** Bars correspond to ΔCt. Surrounded bars correspond to cases *KIT* ^L541^. Others bars correspond to cases *KIT* ^WT^.

Among the 33 samples tested, 6 had Ct_rf_ > 35 and were therefore considered non-informative (4 among these 6 patients had tissue samples fixed in Bouin). The values of Ct_mut_ and Ct_rf_ are presented in the chart (Figure 2) for the 27 evaluable patients. Five patients (19%) had Ct_mut_ < 38 AND Ct_rf_ < 35 and were considered to harbor *KIT*^L541^; 22 patients (81%) Ct_mut_ > 38 AND Ct_rf_ < 35 were classified as *KIT* wild type (*KIT*^WT^) status.

**Figure 3:**
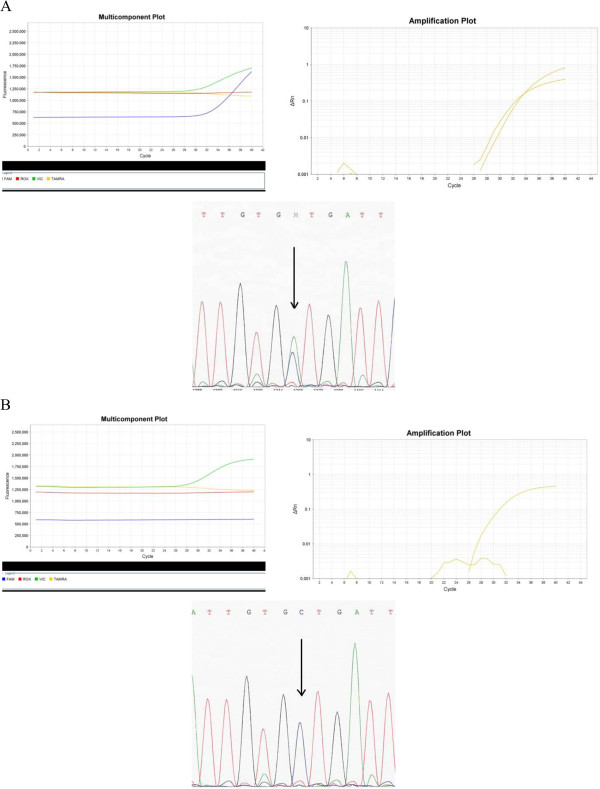
**Determination of** ***KIT*** **status by the 2 methods (sequencing and CAST PCR) for 1 case harbouring** ***KIT*** ^**L541**^ **and 1 case harbouring** ***KIT*** ^**WT**^ **. (A)** Representative multicomponent and amplification plots and sequencing of *KIT* ^L541^ **(B)** Representative multicomponent and amplification plots and sequencing of *KIT* ^WT^

Ten patients of the cohort had double determination of *KIT* status by sequencing and CAST-PCR. Figure 3 presents the determination of *KIT* status by the 2 methods for 1 case harboring *KIT*^L541^ and 1 case harboring *KIT*^WT^.

The clinical characteristics among the 5 patients harboring *KIT*^L541^ are no different from those of the entire cohort. In this subgroup, there are 3 females and 2 males, with a median age at diagnosis of 48 years. The tumor is extra abdominal in 3 cases and located in the abdominal wall in 2 cases with median tumor size of 70 mm [60–189].

Table 2 presents the distribution of objective response according to *KIT* status. Among the 22 patients with *KIT*^WT^ status, 4 patients and 7 patients presented progressive disease at 6 months and 1 year, respectively, compared to no progressive disease at 1 year among the 5 patients harboring *KIT*^L541^. By Chi-2 analysis, the presence of *KIT*^L541^ was not statistically associated with objective response observed at 6 months or at 1 year.

**Table 2:** **Distribution of objective response observed at 6 months and 1 year according to** ***KIT*** **status**

	***KIT*** ^WT^(n = 22)	***KIT*** ^L541^ (n = 5)	Chi 2
Response at 6 months			
CR/PR	3	1	
SD	15	4	
PD	4	0	
			**p = 0,57683407**
Response at 1 year			
CR/PR	2	1	
SD	13	4	
PD	7	0	**p = 0,31614938**

**Figure 4:**
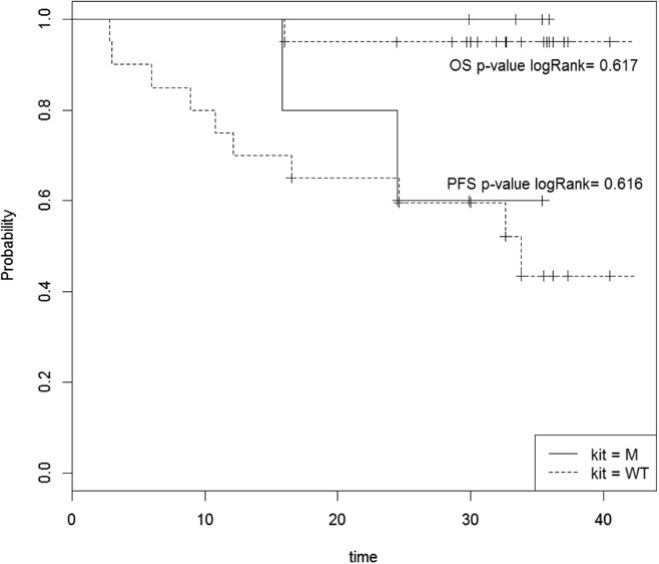
**Log-rank analysis of progression-free survival (PFS) and overall survival (OS) for patients with (M) and without (WT)** ***KIT*** ^**L541**^ **variant in phase II Desminib trial.**

The median PFS of patients harboring *KIT*^L541^ and *KIT*^WT^ is 29.9 and 24.5 months (p = 0.616), respectively and the median OS is not reached, for either group (Figure 4).

## Discussion

The identification of a reliable biomarker to predict treatment efficacy would be useful for the management of AF patients. The possibility that *KIT*^541^ status predicts response to imatinib in AF had been suggested by previous single case studies. In 2010, we failed to precisely determine the biological mechanisms involved in this efficacy but suggest, as others, a possible role of KIT exon 10 M541L variant in the sensitivity of AF to imatinib [14]. Our conclusions were limited by the small cohort analyzed (10 patients), mainly due to the difficulty in extracting sufficient quality and quantity DNA material from FFPE samples to perform sequencing. Taking advantage of technological improvements, this biomarker could be tested in 2012 in the Desminib phase II trial designed to evaluate the activity of imatinib for patients with AF not amenable to local treatment.

Quantitative PCR (qPCR) technologies are developing quickly, sustained by their simplicity to generate robust data. It has already been established that qPCR methods present several advantages, compared with classical sequencing [17]. The use of Taqman-minor-groove-binder (Taqman-MGB) technology is more efficient and more accurate than sequencing. Its selectivity ranges from 1 to 10% according to the level of fragmentation of DNA (25-30% for sequencing). It is an easy one-step method, fast, requiring only basic expertise and less than 2 fold more expensive than sequencing.

Because of these numerous advantages, publications using this method are increasing. The “MIQE précis” (minimum standard guidelines for fluorescence-based quantitative real-time PCR experiments) were applied to the present study to ensure its quality [18]. The superiority of qPCR methods on classical sequencing has been especially established in cases of poor quality FFPE-DNA. Fixation, embedding and extraction methods may lead to the degradation and fragmentation of nucleic acid, but FFPE remains the most frequent storage condition of tissue samples. qPCR methods use small amplicon size to partially by-pass this problem of fragmented DNA which is why we chose to use the qPCR method in our study based on FFPE samples embedded 7 to 15 years earlier.

It has already been demonstrated that CAST-PCR allows efficient amplification of nucleic acids from FFPE samples [19]. It was adopted to analyze FFPE samples from the Desminib trial since AFs have a low cellular density, and with DNA quality deteriorated by FFPE conditions of preservation. Moreover, AF tissues are characterized by extracellular fibrous matrix known to inhibit PCR reactions. Indeed, the efficiency of the CAST-PCR method was confirmed for the FFPE samples of AF with the validation of CAST-PCR results by classical sequencing of 10 cases, allowing us to determine the KIT exon 10 mutational status in 33 cases.

Statistical analyses failed to demonstrate any correlation between *KIT*^541^ status and objective response at 6 and 12 months or survival while undergoing treatment with imatinib. However, it is important to note that no patient with tumor harboring *KIT*^L541^ presented progressive disease at 6 or 12 months, as compared to 4 and 7 patients presenting progressive disease at 6 and 12 months, respectively, in the *KIT*^WT^ cohort. Based on these results, *KIT*^L541^ was not found to be a predictive biomarker for the efficacy of imatinib, but it must be noted that the power of the study remained limited by the small size of the cohort; a similar study is ongoing in the lab on GIST samples.

Multiple activating *KIT* mutations have been described in the extra and intra cellular domain of the receptor. Several mutations have been described in the transmembrane domain encoded by exon 10, and one recently reported was associated with response to imatinib [20]. The predictive value of a Single Nucleotide Polymorphism (SNP) has not been reported, even though several reports show that the *KIT*^L541^ variant may provide a positive signal in different diseases. Foster and Rocha independently reported the presence of *KIT*^L541^ in 5 patients with mastocytosis, in 2 pairs of twins (children) and in 1 adult, respectively [21, 22]. Foster combined this clinical observation with *in vitro* analysis demonstrating that FDC-P1 cells transfected with *KIT*^L541^ showed an enhanced proliferative response, only to low levels of stem cell factor (SCF) (≤6.25 ng/ml), but did not confer factor independence. *KIT*^L541^ cells were also around 2 fold more sensitive to imatinib than those expressing *KIT*^WT^. Inokuchi et al. explored the role of *KIT*^L541^ in chronic myelogenous leukemia (CML) patients [23]. They first observed a statistically significant higher frequency of the variant in patients (6/80, 7.5%) than in healthy controls (1/68, 1.5%: p < 0.05, Fisher’s exact test), partly due to newly occurring mutations at blastic crises. They also performed *in vitro* experiments on *KIT*^L541^ Ba/F3 cells showing that tyrosine kinase activation and proliferative response of *KIT*^L541^ cells were slightly higher than *KIT*^WT^ in medium containing 0.1 ng/ml SCF. Krüger *et al.* were not able to confirm these results screening 102 CML patients and 166 healthy controls in a Caucasian population [24]. They found no differences in the allele frequencies for *KIT*^L541^ variant among patients (16/102, 15.7%) and controls (26/166, 15.7%). Grabellus *et al.* also detected no difference in genotype frequency of *KIT*^L541^ in cases of AF (7/42, 16.7%) compared with healthy population (26/166, 15.7%) [25]. As expected for a SNP, they also detected *KIT*^L541^ variant in adjacent non-neoplastic tissue (muscle) in 4 out of 4 *KIT*^L541^ positive cases with normal tissue available. The authors concluded that *KIT*^L541^ represented a SNP devoid of functional importance with no role in tumorigenesis in AF.

## Conclusion

Our results confirm the efficiency of CAST-PCR as a reliable qPCR method to determine mutational status. Our analyses do not support a predictive value of *KIT*^L541^ in efficacy of imatinib for patients with AF. The significance of the *KIT*^L541^ variant remains unclear.
